# Gender Differences in the Perceived Behavior of Narcissistic Leaders

**DOI:** 10.3389/fpsyg.2022.809193

**Published:** 2022-03-18

**Authors:** Emma J. G. Van Gerven, Annebel H. B. De Hoogh, Deanne N. Den Hartog, Frank D. Belschak

**Affiliations:** Amsterdam Business School, University of Amsterdam, Amsterdam, Netherlands

**Keywords:** leader narcissism, gender, inconsistent leader behavior, LMX, follower task performance

## Abstract

Although narcissists often emerge as leaders, the relationship between leader narcissism and follower performance is ambiguous and often even found to be negative. For women, narcissism seems especially likely to lead to negative evaluations. Since narcissists have the tendency to be impulsive and change their minds on a whim, they may come across as inconsistent. We propose “inconsistent leader behavior” as a new mechanism in the relationship between leader narcissism and follower performance and argue that leader gender plays an important role in whether narcissistic leaders are perceived as inconsistent. Specifically, we expect leader narcissism to have a negative relationship with follower performance through perceived inconsistent leader behavior, especially for female leaders. Thus, we examine leader gender as a personal factor moderating the relationship between narcissism and perceived inconsistent behavior. Also, as perceived inconsistency is likely less problematic when a good relationship exists, we examine leader–member exchange (LMX) as a contextual condition moderating the relationship between leader behavior and follower performance. We test our moderated mediation model in a multi-source study with 165 unique leader–follower dyads. As expected, leader narcissism was positively related to perceived inconsistent leader behavior, and this relationship was stronger for female leaders. Inconsistent leader behavior was negatively related to follower performance, but only when LMX was low. Our research highlights that perceived behavioral inconsistency can be problematic and—for female leaders—provides an explanation of the negative relation of leader narcissism with follower performance and of the inconsistencies in evaluations of narcissistic leaders’ effectiveness.

## Introduction

Narcissism has attracted attention in leadership research for over 20 years. In line with the higher leadership ratings narcissists often receive, they tend to emerge as leaders (Brunell et al., 2008; Nevicka et al., 2011a) and are relatively overrepresented in organizations (Grijalva et al., 2015). However, once narcissists occupy a leadership position, overall they do not seem to be more effective than their less-narcissistic counterparts (Grijalva et al., 2015) and despite initially making a leaderlike impression, over time they are often regarded negatively (Lubit, 2002). This may be due to the characteristics inherent in narcissism.

Narcissistic characteristics overlap with typical agentic traits, such as arrogance (Campbell et al., 2002), exploitativeness, egocentrism (Sedikides and Campbell, 2017), opportunism (Konrath et al., 2016), and impulsivity (Vazire and Funder, 2006; Malesza and Ostaszewski, 2016). These characteristics of narcissism imply an element of irrationality and unpredictability, suggesting that narcissistic leaders are more likely to be perceived as displaying inconsistent leader behavior. Inconsistent leader behavior is behavior that is perceived by followers as varying across situations in erratic and seemingly random ways. These leader behaviors are difficult to predict as they often appear to not fit the situation or differ from previous behavior in a similar situation.

Research has shown that gender impacts the evaluation of characteristics and behaviors, such that men are perceived differently than women depending on the socially expected and accepted sex role behavior (Rudman and Phelan, 2008). Several of the characteristics of narcissists do not fit with the characteristics typically associated with women. For instance, narcissists’ dominant and self-promoting (agentic) behavior is likely to clash with the communal female gender stereotype (e.g., Rudman, 1998). Though this clash can lead to an increase in perceived competence, at the same time it likely leads to a decrease in likeability which is called the backlash effect (Rudman, 1998). The backlash effect explains negative outcomes of incongruency with gender stereotypes, especially for women. In line with literature on the backlash effect, women have been found to be penalized for displaying dominance (Grijalva et al., 2015). For example, agentic behavior by women is positively related to hiring discrimination (Rudman and Glick, 2001; Phelan and Rudman, 2010) and negatively impacts voting preferences, whereas no such relationship exists for men (Okimoto and Brescoll, 2010). Furthermore, people assign less status and lower salaries to women expressing anger as compared to men expressing anger (Brescoll and Uhlmann, 2008). Specifically for a leadership context, gender has been found to impact the relationship between leader narcissism and perceived leader effectiveness where female narcissistic leaders are rated as less effective than male narcissistic leaders (De Hoogh et al., 2015). Previous research has also demonstrated that perceivers encode leader behavior in relation to leader gender (Scott and Brown, 2006; Sczesny et al., 2006). However, to our knowledge, the mechanisms underlying gender differences in the evaluation of narcissistic leaders are not yet clear. Here, we propose that inconsistent leader behavior forms a mechanism through which leader narcissism is negatively related to outcomes and that this will be exacerbated for female leaders.

Inconsistent behavior reflects behavior that typically relates to impulsivity and opportunism which are agentic traits (as they both reflect power and selfishness as typical features of agency) that are linked to narcissism (e.g., Jonason and Fletcher, 2018). Impulsivity is characterized by being rash and unpredictable (e.g., Dickman, 1990; Bari and Robbins, 2013). Opportunism is related to efforts to gain an advantage from a situation, often at the expense of others (Wong et al., 2005). Considering that men are expected to display dominant and agentic behavior (Eagly et al., 1981) and are stereotypically thought to be high on impulsiveness (Löckenhoff et al., 2014), displaying inconsistent behavior is congruent with the masculine stereotype. When men display agentic and inconsistent behavior, this may thus be interpreted as a display of power rather than erratic behavior.

Women, on the other hand, are expected to act according to rules and norms and to not display divergent behavior, such as agentic behavior (Eagly et al., 1981). Drawing on Sherif and Hovland’s (1961) classic judgment model we propose that the negative aspects of narcissism in terms of being divergent and unpredictable are discrepant from people’s stereotypes about women and thus more salient when evaluating the behavior of female narcissistic leaders. For women, showing agentic inconsistent behavior may come across as erratic and negatively stands out. This behavior for females is highly visible, whereas for male leaders being unpredictable and impulsive is congruent with the expected (agentic) sex role behavior and will stand out less. We thus expect that the effects of narcissism on the perception of inconsistent leader behavior are contingent on leader gender.

**Figure 1:**
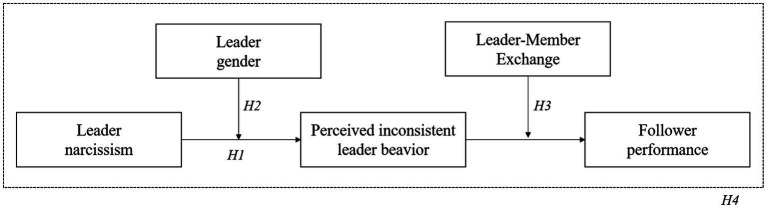
Proposed moderated mediation model.

The perception of inconsistent leader behavior in turn negatively affects follower performance as it acts as a stressor that is likely to deplete followers’ energetic resources (Burger and Arkin, 1980). Previous research suggests that followers’ response to leader behavior is influenced by the quality of the leader–follower relationship, often referred to as leader–member exchange (LMX). According to LMX theory, leaders do not treat every subordinate the same, different types of relationships develop between leaders and followers, and the quality of these relationships can range from low to high (e.g., Liden et al., 1997). Followers in a high-quality relationship have higher trust in their leader and are more committed to the leader. This makes them more open to social (leader) influence and implies they respond more favorably to their leader’s behaviors than followers in a low-quality relationship (Piccolo and Colquitt, 2006; Michel and Tews, 2016). In line with this, we argue that the effects of perceived inconsistent leader behavior on follower performance are dependent on the quality of the relationship between leader and follower. For followers in a high-quality LMX, where best intent of the leader is assumed and trust in the leader is high, the negative effects of perceptions of inconsistent leader behavior on followers will be reduced compared to a low-quality LMX relationship. We thus test a (first-stage and second-stage) moderated mediation model that may help to clarify the link between leader narcissism and follower performance and the effect of gender on this relationship (see Figure 1).

With this paper, we aim to contribute to several literatures. First, we add to the research on gender differences by investigating the effect of gender on the perception of narcissistic leader behavior. In doing so we contribute to the understanding of why narcissism and related behaviors are differentially perceived for men and women. Second, explanatory variables for the negative impact of narcissism on follower performance have rarely received attention to date (for an exception, see Nevicka et al., 2011b). Here, we introduce the concept of inconsistent behavior to the literature on narcissism and identify it as a mechanism through which leader narcissism may relate to follower performance. Third, we answer a call of leadership scholars who have emphasized the need for theory development on behavioral inconsistency (e.g., Simons, 2002), which has only recently started to receive (limited) research attention (e.g., Dineen et al., 2006; Zhang et al., 2015). Finally, we propose LMX as a contingency variable to mitigate the negative effects of perceived inconsistent leader behavior on follower performance hereby adding to the literature showing the moderating effects of LMX on followers’ reactions to their leaders’ behavior.

### Leader Narcissism

Narcissism describes a personality trait that involves a lack of empathy, inflated self-esteem, and a need for admiration (Miller and Campbell, 2008). The lack of empathy that characterizes narcissists implies a disrespect and disregard of others (Konrath et al., 2016) and over time this often creates difficulties in maintaining close relationships (Campbell and Foster, 2002). Their inflated self-esteem biases narcissists’ self-perceptions by making them dream about personal success, glory, and power, and by stimulating them to see themselves as superior to others (Paulhus and Williams, 2002; Rosenthal and Pittinsky, 2006). Narcissists have high levels of confidence and optimism and seek power and authority over others (Raskin and Terry, 1988; John and Robins, 1994). Narcissists also view themselves as very intelligent, special, and unique and have a tendency to be arrogant (Raskin and Terry, 1988; Campbell et al., 2002; Judge et al., 2006). Narcissists’ self-view, however positive, is unstable (Baumeister et al., 2000). They need admiration and constant reaffirmation of their self-implied superiority (Rosenthal and Pittinsky, 2006), which is why they engage in social displays of ability, act in ways to reinforce their superiority, and favor bold actions that attract attention (Chatterjee and Hambrick, 2007; Smith and Webster, 2018).

Research shows that people scoring high on narcissism score low on ethics (Brown et al., 2010). Narcissism forms a predictor of counterproductive work behavior (Grijalva and Newman, 2015) and lying (Giammarco et al., 2013). Narcissists are erratic and often act impulsively (Jones and Paulhus, 2011), and they are unable to learn from mistakes (Campbell et al., 2004) or to react to negative feedback in an appropriate way (Barry et al., 2006). In what follows, we focus on a so far under-researched aspect of narcissism and propose that the impulsivity of narcissists can lead to narcissistic leaders being perceived as displaying inconsistent leader behavior, especially for female leaders, which might explain the negative relationship between leader narcissism and follower performance.

### Narcissism and Gender

Research has shown that the same characteristics are evaluated differently when displayed by men and women, depending on social expectations and accepted role behavior (Rudman and Phelan, 2008). Social role theory suggests that women are expected to be communal (helping, understanding) while men are expected to be agentic (dominant, arrogant; Eagly, 1987). These gender-related expectations are also found for leaders. Previous research has demonstrated that perceivers encode leader behavior in relation to leader gender (Scott and Brown, 2006; Sczesny et al., 2006). For example, women are more likely to be expected to have a servant leadership style, whereas men are more likely to be expected to have an authoritarian style (Hogue, 2016).

Many narcissistic characteristics overlap with agentic traits (Campbell et al., 2002), which implies that narcissism is more in line with stereotypical masculine traits as compared to feminine traits. The dominant and self-promoting behavior that is typical for narcissists, does not match the communal leadership style that is expected of female leaders (e.g., Rudman, 1998). Yet, being incongruent with one’s gender role might lead to negative evaluations, resulting in a backlash effect for agentic female leaders (e.g., Rudman, 1998; Eagly et al., 2000). Indeed, narcissistic leaders are evaluated negatively when they are women, but not when they are men (e.g., De Hoogh et al., 2015). Here, we build on this work on narcissism and gender. Based on the judgment model of Sherif and Hovland (1961) we argue that behavior that is incongruent with one’s stereotype is more salient because of the contrast between the behavior displayed and the behavior expected based on gender role expectations and is therefore perceived more negatively. While prior research has focused particularly on the dominance and assertiveness of narcissistic leaders (Rosenthal and Pittinsky, 2006), other narcissistic characteristics have received less attention. Here, we explore narcissists’ unpredictability and inconsistency as under-researched characteristics.

### Inconsistent Leader Behavior

Previous work in the field of leadership has often described leadership styles as stable and constant, suggesting that leaders typically display one type of behavior (Hannah et al., 2014). Even though early research based on contingency theory (e.g., Fiedler, 1967) already pointed out that leader behavior may differ from situation to situation (Utecht and Heier, 1976), only recently have researchers started to investigate the effects of leaders displaying multiple types of leader behavior (e.g., Johnson et al., 2012; Lanaj et al., 2016).

Most theories that address multiple leader behaviors, like contingency theory, leader versatility, and flexible leadership (Fiedler, 1967; Kaplan and Kaiser, 2003; Yukl and Mahsud, 2010), focus on leader’s display of varying behavior in order to adapt to specific situational or personal demands. Here, we argue that leaders may also engage in varying behavior that is not *per se* perceived to be adjusted to a specific situation. For example, leaders might be approachable one moment and not the next without a clear reason. Differences or changes in leader behaviors that occur at different moments or in different spaces may have their roots in other contexts (e.g., meetings with top management that followers have no notion of may cause a change in actions), personality traits of the leader (e.g., impulsivity, instability), strategic intent (e.g., self-centered), or (lack of) competency. As followers lack knowledge of the source of the unpredictability, followers may perceive such variation in leader behavior as unpredictable, erratic, and inconsistent, and this may negatively impact followers and organizations, for instance, by undermining trust in the leader, distracting followers, and causing them stress.

So far, only few researchers have looked into such potentially negative behavioral variability. For example, Dineen et al. (2006) studied the effect of leader’s inconsistency between words and actions on follower organizational citizenship behavior. Organizational citizenship behavior (OCB) can be defined as behavior that is not part of an employee’s formal tasks, such as voluntarily providing assistance to colleagues or promoting the organization (e.g., Smith et al., 1983; Organ, 1988) or as “performance that supports the social and psychological environment in which task performance takes place” (Organ, 1988, p. 95). OCB is an important construct in organizational research as it is related to measures of organizational effectiveness (Podsakoff et al., 2009). In two separate field samples, Dineen et al. (2006) found that leader’s consistency between words and actions is positively related to follower OCB. This suggests word-deed misalignment (“not walking your talk”) may have negative consequences, especially considering the positive effects of OCB on organizational effectiveness. Also, another study suggested that when leaders who display ethical leader behavior are also seen to display passive leader behavior, this reduces the positive effects of the ethical behavior. Specifically, the findings show that passive behavior weakens the negative effect of ethical leader behavior on follower burnout (Vullinghs et al., 2020), again suggesting that varying leader behavior may have adverse consequences.

In this paper, we argue for an overarching type of inconsistent leader behavior, which is not limited to inconsistency between values and behavior or varying between different leadership styles. Leaders that display inconsistent leader behavior show different behavior in similar situations (e.g., stressing the importance of a specific goal 1 day, whereas the next day another goal is emphasized as most important) or treat similar followers differently (e.g., showing appreciation for the achievement of one follower, but not for similar achievements of another), which makes their behavior hard to predict for followers. Given that leaders have considerable power over organizational processes and outcomes, inconsistent leader behavior may be particularly impactful. Not being able to predict the behavior of their leader is likely to be cognitively and emotionally taxing for followers and thus may deplete resources and distract followers from their core tasks as they constantly feel the need to monitor their leader to make sense of the inconsistent behavior and understand the leader’s intentions.

Narcissists are often described as opportunistic (Wink, 1991; Konrath et al., 2016) and impulsive (Vazire and Funder, 2006). On average, they score low on both empathy (Ames and Kammrath, 2004) and agreeableness (Paulhus, 2001). Narcissists are also characterized by an extreme impulsivity and *ad hoc* emotional reactivity and display more day-to-day variability and extremity in their emotions than less-narcissistic individuals (Emmons, 1987; Rhodewalt et al., 1998). Moreover, narcissists use other people to further their own goals (Campbell et al., 2005; Rosenthal and Pittinsky, 2006; Sedikides and Campbell, 2017; Den Hartog et al., 2020). They believe they deserve more than others and have a high sense of entitlement (Campbell et al., 2004). People with a sense of entitlement may see their own motivation as sufficient to act, thereby disregarding others’ ideas, needs, and objections. They focus on acting on their desires, including ones that others might find rather questionable (Hofmann et al., 2012). This suggests that narcissists will easily alternate between behaviors depending on what they feel is best for them, or on a whim based on what they feel like in the moment. We propose this may lead to them being perceived by others as inconsistent.

Indeed, research has found narcissists to take advantage of specific situations. They are, for instance, more likely to engage in prosocial behavior when this behavior is highly visible than when no one can see it (Konrath et al., 2016). Moreover, narcissism is positively linked to impulsivity (Casillas and Clark, 2002), independent behavior, and lower ability to delay gratification (Vazire and Funder, 2006). Narcissists experience less conflict when acting on their desires (Hofmann et al., 2012). Moreover, research suggests that narcissists may strategically act in ways that imply they do not inhibit their urges and may intentionally engage in inconsistent, volatile behavior to convey a sense of power (Hart et al., 2017). Indeed, research shows that unpredictability may increase the (perceived) power of leaders (e.g., Sullivan et al., 2010; Van Kleef et al., 2011, 2012). We thus hypothesize:

*Hypothesis 1*: Leader narcissism is positively related to perceived inconsistent leader behavior.

### Leader Gender and Perceived Inconsistent Behavior

As noted, the same traits and behavior can be evaluated differently for men and women. The Sherif and Hovland (1961) judgment model suggests that stereotype-inconsistent actions are contrasted with gender role expectations. Behavior that is not expected is noticed more easily because of this contrast effect. For example, because women are expected to be more understanding and kind than men, a man is more likely to be noticed when comforting his child than a woman doing the same thing. Many studies found evidence for such stereotype-based contrast effects (e.g., Rudman and Phelan, 2010). We argue that such contrast effects with regard to gender stereotype may also affect perceptions of narcissistic leaders’ inconsistent behavior.

Specifically, men are socialized to be more aggressive, autonomous, and bold, while women exhibit more conformity and self-discipline (Low, 1989). Also, research has consistently found gender differences in self-control with women exhibiting higher levels of self-control than men (LaGrange and Silverman, 1999). Whereas men generally score higher than women on self-reported emotional stability (Costa et al., 2001), evidence indicates an opposite pattern when it comes to stereotypes about this trait: men are on average stereotyped to be higher in impulsiveness than women (Löckenhoff et al., 2014). Drawing on Sherif and Hovland’s (1961) stereotype-based judgment model we argue that perceivers expect less impulsive and unpredictable behavior from female leaders. Based on gender role expectations agentic, inconsistent actions stand out more for women than for men. These behaviors will thus be more salient when evaluating female narcissistic leaders than when evaluating male narcissistic leaders. Narcissists feel the power to do whatever they want, change their mind on a whim, and act on their impulses, which we hypothesize is more accepted, stands out less, and is less likely to be perceived as inconsistent for male leaders. In contrast for women, such impulsive behavior runs counter gender stereotypes and stands out compared to the expected agreeableness and thus such behavior is perceived as more inconsistent.

*Hypothesis 2*: Leader gender moderates the relationship between leader narcissism and perceived inconsistent leader behavior, such that female narcissistic leaders are perceived as displaying more inconsistent behavior than male narcissistic leaders.

### Inconsistent Leader Behavior and Leader–Member Exchange

Inconsistent leader behavior pertains to showing varying behavior in similar situations, which makes it hard for followers to predict how an inconsistent leader will act. Prior research has suggested that inconsistent behavior may indeed play an important role in increasing experiences of unpredictability (O’Driscoll and Beehr, 1994; De Cremer, 2003). Predictability is valued very much by followers, and unpredictability is typically experienced as a strong stressor (e.g., Monat et al., 1972). For example, followers rate their leaders as more effective and more credible when they are able to predict their behavior (e.g., Johnson et al., 2012). Followers even prefer constant abuse over unpredictable abuse (Matta et al., 2017). A lack of perceived control over a situation and predictability are found to be related to motivational losses due to feelings of helplessness and related declines in performance (Burger and Arkin, 1980). In addition, unpredictable behavior of the leader is a stressor that is likely to deplete followers’ resources and to distract their attention away from their core tasks. We therefore believe that perceived inconsistent leader behavior is negatively related to follower performance.

In previous research, LMX has been studied as an important factor influencing the effects of leader behaviors on followers (e.g., Schriesheim et al., 1998). According to Smircich and Morgan (1982), perceptions of and reactions to leadership are based on the interactions between leaders and followers. The quality of these interactions and the nature of the relationship between leader and follower determines the extent to which followers decide to resist the influence attempts of leaders or be open to them. In this sense LMX functions as “an anchor and context” (Lind, 2001, p. 73) for followers for interpreting and evaluating their leader’s behavior. Indeed, findings suggest that the quality of the relationship between leaders and their followers defines the reaction of followers to leader behavior, where followers in a high-quality relationship have a more positive attitude toward their leader and assume their leader wants what is best for them. Followers in low-quality relationships, on the other side, have lower trust in their leader (Piccolo and Colquitt, 2006; Michel and Tews, 2016).

We study LMX as potentially having a buffering effect, where high levels of LMX might prevent a strong negative effect of perceived inconsistent leader behavior on follower performance. We argue that high-quality LMX makes followers more lenient toward their leaders (Michel and Tews, 2016), and we propose that perceived inconsistent leader behavior may then also have a less negative impact on follower performance under high-LMX leaders. For instance, followers might attribute perceived inconsistent leader behavior to the circumstances as they assume good leader intentions, or they may assume that there must be a good reason for the change in leader behavior that they might not know of. This logic also suggests that low-quality LMX might actually strengthen the negative relationship between perceived inconsistent leader behavior and follower performance as followers are likely more sensitive to and subsequently react more negatively to this type of behavior when feeling less connected to their leader. Thus, we expect:

*Hypothesis 3*: LMX moderates the relationship between perceived inconsistent leader behavior and follower performance, such that the negative effect is weaker when LMX is high as compared to when LMX is low.

Overall, we expect that the indirect relationship between leader narcissism and follower performance *via* perceived inconsistent leader behavior is a function of leader gender (first-stage) and LMX (second-stage).

*Hypothesis 4*: Leader narcissism is related to follower performance *via* a conditional indirect effect, such that the negative indirect effect of leader narcissism on follower performance is strongest for female leaders with a low-quality relationship with their follower.

## Materials and Methods

### Sample and Procedure

We tested our research model in a multi-source field study on a sample of 165 unique leader–follower dyads (i.e., 165 leaders and one follower for each leader, resulting in 165 followers) who worked in different organizations and across different industries. Our sample size is similar to that of samples used in previous studies looking at similar topics and models with the same amount of complexity (e.g., De Hoogh et al., 2015). Dyads were approached through contacts of students of a Dutch university and, if they agreed to participate, an email invitation to an online survey was sent. Confidentiality and the voluntary nature of participation were stressed in the accompanying message. To ensure anonymity, participants received a unique code to match the surveys. Participants could choose to complete the survey either in English or in Dutch. During data collection, reminders were sent to participants to increase the response rate. Most leaders were male (61.8%), the mean age was 41.98 years (SD = 11.62, 1 missing value). On average, leaders had worked for their current organization for 11.13 years (SD = 9.27, 9 missing values) and had worked with this specific follower for 3.86 years (SD = 4.63, 15 missing values). Most followers were female (50.3%), the mean age was 35.07 years (SD = 12.86). On average, followers had been working at their current organization for 7.98 years (SD = 9.23, 21 missing values).

### Measures

Leaders rated their followers’ performance and their own personality. Followers rated leader behavior and LMX. All variables were measured using a 7-point Likert-scale ranging from 1 (*strongly disagree*) to 7 (*strongly agree*).

#### Narcissism

Leaders filled in the 13-item version of the Narcissistic Personality Inventory (NPI-13; Gentile et al., 2013). Example items are: “I like having authority over others,” and “I will usually show off if I get the chance.” Coefficient alpha was 0.84.

#### Inconsistent Leader Behavior

As the concept of inconsistent leader behavior has received little attention in the leadership literature to date, we used a relatively new scale developed by Van Gerven et al. (2021) for measuring Inconsistent Leader Behavior (ILB). Four items were generated by Van Gerven et al. (2021) that matched the definition of inconsistent leader behavior and aimed to capture a one-dimensional focus on leader behavior that is perceived by followers as unpredictable and erratic and the authors provide validity information for this scale from multiple samples. Cronbach’s alpha of this four-item scale was 0.87. For the full set of items see Table 1.

**Table 1:** Inconsistent Leader Behavior items.

Item number	Item
1	My supervisor behaves alternately.
2	My supervisor is inconsistent in his/her behavior.
3	My supervisor is hard to predict.
4	My supervisor behaves differently in comparable situations.

#### Leader–Member Exchange

Leader–member exchange was measured using the 8-item scale by Liden et al. (1993). Example items are: “My supervisor would be personally inclined to use his/her power to help me solve problems in my work,” and “My supervisor understands my problems and needs.” Coefficient alpha was 0.87.

#### Task Performance

Follower performance was measured using a five-item scale filled out by the leader (Williams and Anderson, 1991). Example items are: “My employee adequately completes assigned duties,” and “My employee meets formal performance requirements of the job.” Coefficient alpha was 0.87.

#### Control Variables

As the negative effects of narcissism might grow over time (Paulhus, 1998), we included tenure with the leader (in years) as control variable. We also checked whether survey language made a difference. Tenure did not significantly alter the variables or relationships in our study. Analysis conducted with language of the survey as a control also produced the same pattern of results. To conserve statistical power we therefore report the results without these control variables in what follows (e.g., Becker, 2005).

#### Measurement Model

A confirmatory factor analysis was conducted to determine whether the data conformed to the assumption that each of the proposed latent variables represents a separate construct. We randomly combined subsets of narcissism items to create three parcels of items. We did this only for the well-established and validated narcissism measure as this sufficiently reduced the sample size to parameter ratio, for the other variables we retained the single items and did not use parceling. Results for the measurement model indicated that the four-factor model fitted the data well, *χ^2^*(164, 165) = 310.217, *p* < 0.01, CFI = 0.915, TLI = 0.902, RMSEA = 0.074, SRMR = 0.059. Two alternative models, one in which the items of leader narcissism and inconsistent leader behavior were merged into one factor, *χ^2^*(167, 165) = 532.806, *p* < 0.01, CFI = 0.788, TLI = 0.759, RMSEA = 0.115, SRMR = 0.092, Δ*χ^2^*(3) = 222.589, *p* < 0.001, one in which the items of inconsistent leader behavior and leader–member exchange were merged into one factor, *χ^2^*(167, 165) = 541.421, *p* < 0.01, CFI = 0.783, TLI = 0.753, RMSEA = 0.117, SRMR = 0.086, Δ*χ^2^*(3) = 231.204, *p* < 0.001, exhibited significantly poorer fit. We also compared the four-factor model with a two-factor model with the items of leader narcissism and follower performance (both rated by the leader) in one factor, and inconsistent leader behavior and leader–member exchange (both rated by the follower) merged into the second factor. The four-factor model showed a significant better fit over the two-factor model, *χ^2^*(169, 165) = 785.379, *p* < 0.01, CFI = 0.643, TLI = 0.598, RMSEA = 0.149, SRMR = 0.125, Δ*χ^2^*(5) = 475.162, *p* < 0.001.

## Results

### Correlations

Table 2 presents the means, standard deviations, correlations, and reliabilities of the variables. Leader narcissism was positively correlated with perceptions of inconsistent leader behavior (*r* = 0.21, *p* = 0.006) and negatively with LMX (*r* = −0.15, *p* = 0.049). Perceptions of inconsistent leader behavior were negatively correlated with follower performance (*r* = −0.16, *p* = 0.042) and LMX (*r* = −0.46, *p* < 0.001). Finally, LMX was positively correlated with follower performance (*r* = 0.22, *p* = 0.005).

**Table 2:** Means, standard deviations, and correlations (Cronbach Alphas on diagonal).

	*M*	SD	1	2	3	4	5	6	7
1. Follower gender	1.50	0.50							
2. Leader gender	1.38	0.49	0.41^**^						
3. Leader tenure with follower	3.86	4.63	−0.02	−0.18^*^					
4. Leader narcissism	3.82	0.86	−0.08	0.00	−0.07	(0.85)			
5. ILB	2.66	1.35	−0.03	0.05	−0.05	0.21^**^	(0.87)		
6. LMX	5.40	0.91	0.09	0.08	0.08	−0.15^*^	−0.46^**^	(0.87)	
7. Follower performance	5.95	0.72	0.08	−0.05	−0.01	0.00	−0.16^*^	0.22^**^	(0.87)

*N* = 165 (150 for tenure). Tenure in years. Men are coded as 1, women are coded as 2. ILB, Inconsistent leader behavior, and LMX, Leader–member exchange.

* *p < 0.05;*

** *p < 0.01*.

### Hypothesis Testing

To test the hypotheses, we used the PROCESS macro (model 21, version 3.4, Hayes, 2013) in SPSS to conduct our analyses. All variables were mean centered prior to analyses (Aiken and West, 1991). The first step of this analysis examines the main effect of leader narcissism on inconsistent leader behavior. Hypothesis 1, leader narcissism is positively related to perceptions of inconsistent leader behavior, was supported. The results showed a significant main effect of leader trait narcissism on follower perceptions of inconsistent leader behavior (*B* = 0.34, SE = 0.12, *t* = 2.91, *p* = 0.004, 95% CI [0.11, 0.58]).

Next, we tested our full moderated mediation model. Hypothesis 2, leader gender moderates the relationship between leader narcissism and perceptions of inconsistent leader behavior, was supported (*B* = 0.59, SE = 0.24, *t* = 2.41, *p* = 0.017, 95% CI [0.11, 1.07]). Female narcissistic leaders were perceived to display more inconsistent behavior (*B* = 0.71, *SE* = 0.20, *t* = 3.62, *p* < 0.001, 95% CI [0.32, 1.09]), whereas narcissism was not related to perceptions of inconsistent behavior for male narcissistic leaders (*B* = 0.12, SE = 0.15, *t* = 0.79, *p* = 0.428, 95% CI [−0.17, 0.41]). The moderating effect of LMX on the relationship between perceptions of inconsistent leader behavior and follower performance (Hypothesis 3) was also supported (*B* = 0.09, SE = 0.04, *t* = 2.11, *p* = 0.036, 95% CI [0.01, 0.18]). Perceived inconsistent leader behavior was negatively related to follower performance for leaders with low LMX (1 SD below the mean; *B* = −0.11, SE = 0.05, *t* = −1.94, *p* = 0.054, 95% CI [−0.21, 0.00]), but not for leaders with high LMX (1 SD above the mean; *B* = 0.06, SE = 0.07, *t* = 0.88, *p* = 0.380, 95% CI [−0.07, 0.19]).

**Figure 2:**
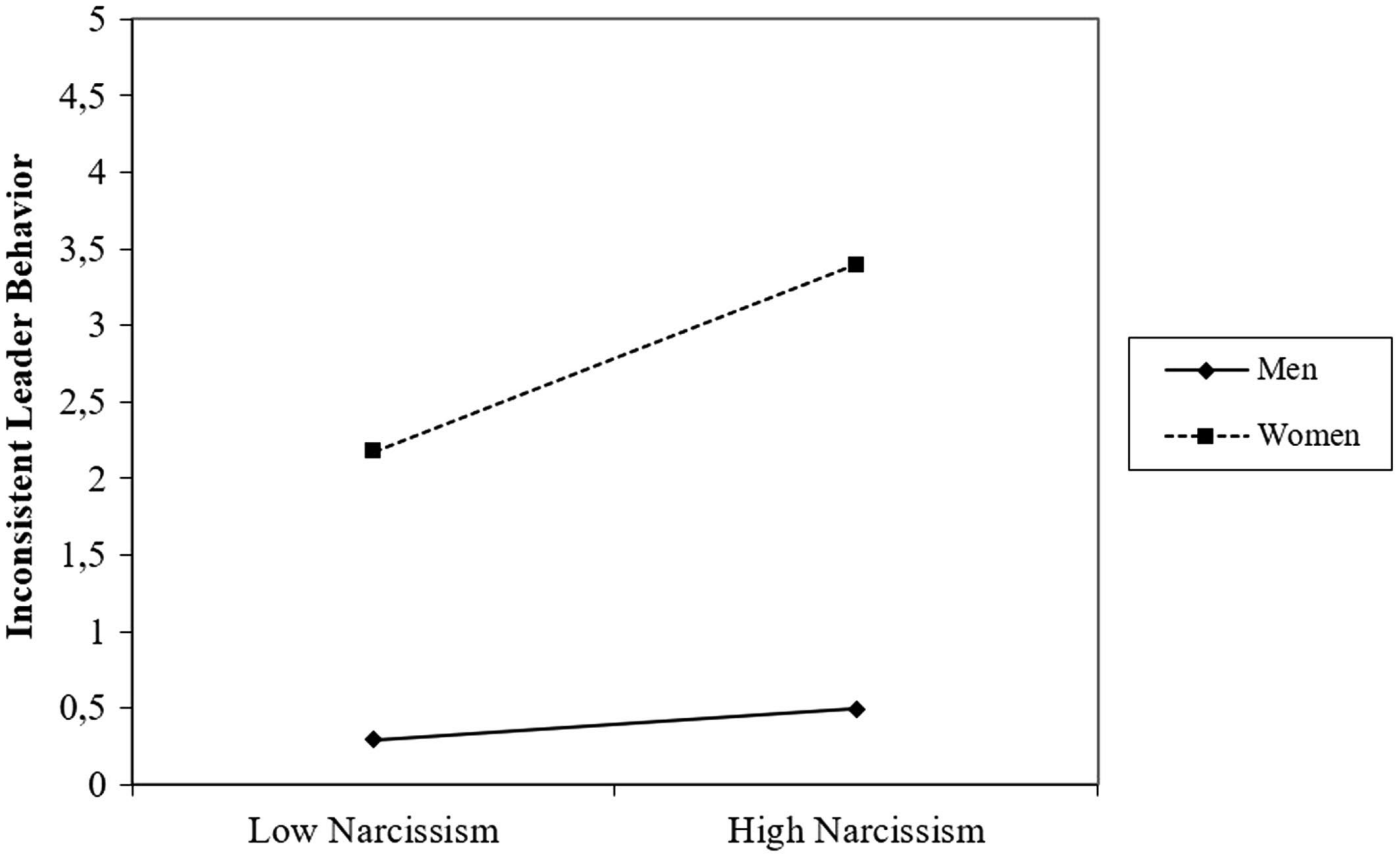
Effect of leader narcissism and leader gender on follower perceptions of inconsistent leader behavior.

**Figure 3:**
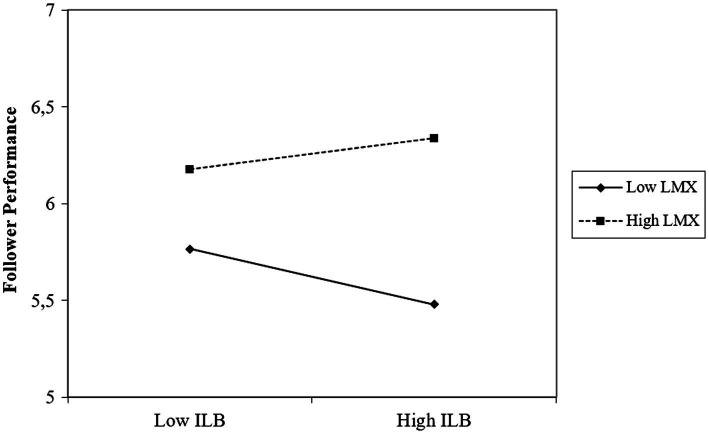
Effects of follower perceptions of inconsistent leader behavior (ILB) and quality of the leader-follower relationship (LMX) on follower performance.

Finally, we found support for a conditional indirect effect of leader narcissism on follower performance *via* perceptions of inconsistent leader behavior moderated by leader gender and LMX (Hypothesis 4) as the index of moderated mediation was significant, which means that the indirect relationship of leader narcissism with follower performance through inconsistent leader behavior was found to be a function of gender and LMX (Index = 0.0537, SE = 0.03, 95% CI [0.001, 0.135]). As predicted, for female leaders with low LMX (1 SD below the mean), leader narcissism was negatively related to follower performance through perceptions of inconsistent leader behavior (*B* = −0.08, SE = 0.04, 95% CI [−0.169, −0.002]). For female leaders with high LMX (1 SD above the mean), the negative relationship between inconsistent leader behavior and follower performance became insignificant and the indirect relationship was no longer there (*B* = 0.04, SE = 0.04, 95% CI [−0.040, 0.138]). For male leaders there was no indirect negative relationship between leader narcissism and follower performance through perceptions of inconsistent leader behavior, both when they had low LMX (1 SD below the mean; *B* = −0.01, SE = 0.02, 95% CI [−0.053, 0.021]) as well as when they had high LMX (*B* = 0.01, SE = 0.01, 95% CI [−0.019, 0.038]), providing further support for Hypothesis 4. See Figures 2, 3 for the interaction effects. Results of the moderated mediation analysis are presented in Table 3.

**Table 3:** Results of the moderated mediation analysis using the PROCESS macro 21.

Predictor	*B*	*se*	*t*	(95% CI)
Inconsistent Leader Behavior
Constant	−0.00	0.10	−0.00	(−0.20, 0.20)
Predictors				
Leader narcissism	0.34^**^	0.12	2.91^**^	(0.11, 0.58)
Leader gender	0.14	0.21	0.67	(−0.27, 0.55)
Leader narcissism ^*^ Leader gender	0.59^*^	0.24	2.41^*^	(0.10, 1.07)
Follower performance
Constant	0.05	0.06	0.85	(−0.07, 0.17)
Predictors				
Leader narcissism	0.04	0.07	0.55	(−0.09, 0.16)
ILB	−0.02	0.05	−0.49	(−0.12, 0.08)
LMX	0.11	0.07	1.50	(−0.03, 0.25)
ILB ^*^ LMX	0.09^*^	0.04	2.11^*^	(0.01, 0.18)
Index ILB	0.0537^*^	0.035		(0.001, 0.135)

*N = 165*.

* *p < 0.05;*

** *p < 0.01*.

## Discussion

In this paper, we aimed to contribute to the narcissism, gender, and the leadership literature by specifying a mechanism through which leader narcissism might influence follower performance, namely, perceived inconsistent leader behavior, and identifying gender as a moderator. Previous work shows that narcissists have the tendency to be impulsive and feel entitled to change their minds on a whim and that narcissistic leaders are evaluated differently based on their gender. To date, an explanation for these gender differences is lacking. To address this, we drew from the literature on narcissism and leadership and proposed a moderated mediation model in which the relationship of leader narcissism with follower performance is mediated by perceived inconsistent leader behavior.

We expected the effect of leader narcissism on perceptions of inconsistent leader behavior to depend on leader gender. Also, we expected the effect of perceptions of inconsistent leader behavior to depend on the quality of the relationship between the leader and the follower. In a multi-source field study, we found support for the expected gender differences. Specifically, we found that leader narcissism was indirectly negatively related to follower performance *via* perceived inconsistent leader behavior, but only for followers of female leaders who experience low LMX. This suggests that a high-quality relationship may act as a buffer for the potential negative effects of narcissism and perceived inconsistent leader behavior, specifically for female leaders.

### Theoretical Implications

Our research contributes to the gender literature by further developing insights into why the effects of leader narcissism and follower outcomes tend to differ between men and women. Specifically, we found that female narcissists are perceived as displaying more inconsistent behavior, and this may be one explanation for inconclusive findings in evaluations of narcissistic leaders. Prior research showed that narcissists are more impulsive (Vazire and Funder, 2006) and opportunistic (Konrath et al., 2016). Here we show how these characteristics seem to translate into female narcissistic leaders being perceived as behaving inconsistently in the leadership role, which is experienced negatively by followers. Our research adds to the stream of research on gender differences and judgment (e.g., Sherif and Hovland, 1961) by showing that the negative aspects of narcissism in terms of being divergent and unpredictable seem to be more salient for women. Such behavior is incongruent with people’s stereotypes about women and thus perceptually contrasted from these stereotypes (see also Manis et al., 1988). For men, agentic behaviors, such as being dominant and erratic, do not seem to be incongruent and do not come with a backlash. However, this backlash effect does happen for women: the contrast with gender expectations does seem to translate into negative evaluations. This new negative and gendered pathway from leader narcissism to follower performance aids in explaining differences in the relationship of narcissism and leader effectiveness for men and women (De Hoogh et al., 2015).

Furthermore, we answer the call for more research into behavioral inconsistency and related constructs (Simons, 2002) by looking into both antecedents and outcomes of perceptions of inconsistent leader behavior. Although varying leader behavior has been studied previously, it was usually studied from a positive perspective (how leaders vary behavior in order to be flexible or adapt to the situation or person), narrowed down to a specific type of inconsistency (e.g., word-deed alignment; Dineen et al., 2006), or focused on displaying two different leadership styles simultaneously (e.g., Vullinghs et al., 2020). Here, we contribute theoretically by showing that perceptions of overall inconsistent behavior form a broad construct that seems to be negatively related to follower performance.

### Managerial Implications

Our research findings show that it is important to pay attention to gender effects. Because agentic traits are to some extent deemed necessary to be able to make it at the top (Eagly and Karau, 2002) researchers have previously focused on potential “buffers” for the effects of non-stereotypical gender behavior. Even in leadership roles, gender differences exist along the communal dimension (Moskowitz et al., 1994) as female leaders show empathy and build relationships more readily than their male counterparts (Fletcher et al., 2000). For female leaders, it seems that being high on agentic traits might be accepted as long as those traits do not conflict with the prescription for women of being kind and displaying communal behavior (Prentice and Carranza, 2002). This suggests that displaying desirable communal behavior might prevent negative outcomes. In our study, we find that indeed female narcissistic leaders can make up for their display of counter stereotypical agentic behavior by forming high-quality interpersonal relationships with subordinates.

Since a high-quality relationship mitigates negative effects of perceptions of inconsistent leader behavior, regardless of the gender of the leader, organizations should think of ways to help leaders to improve the relationships with their followers. Providing support and displaying loyalty and trust characterize a high-quality LMX (Graen and Scandura, 1987; Uhl-Bien and Maslyn, 2003) and explicit attention to supporting followers could therefore help in improving the quality of leader–follower relationships. Furthermore, leaders might provide more rationales for their behaviors because explanations for behavior can positively influence the perception and interpretation of leader behavior by followers (Simons, 2002). Transparency might thus help to minimize problems of inconsistent leader behavior.

Finally, our findings provide valuable insights into the overrepresentation of male leaders and how this might relate to (toxic) workplace cultures. While research on narcissism has established a positive link between narcissism and leader emergence as well as leadership ratings (e.g., Brunell et al., 2008; Nevicka et al., 2011a), our results suggest that particularly men might profit from this. Whereas female narcissistic leaders experience backlash, our findings suggest that narcissism is more readily accepted in male leaders allowing them to occupy leader positions, typically accompanied by power. As leaders form role models for followers, agentic and unpredictable behavior shown by narcissistic male leaders might be seen as acceptable and hence “rub off,” thereby potentially creating a negative culture of inconsistency.

### Limitations and Future Research Directions

Despite its contributions, we recognize that our study has limitations. First, even though we use a multi-source design and focus on leader trait narcissism, a personality characteristic, as our independent variable and behaviors as mediators and outcome variable, our research design was cross-sectional, which means we cannot draw firm conclusions about causality. Also, we used a non-probability sampling method which might limit the generalizability of our findings. Future research should consider studying the variables in an experimental setting and use a more systematic sampling approach.

In future studies, it will be important to investigate the specific mechanisms expected to underly the gender differences, namely, gender role expectations. We do find that female narcissistic leaders are perceived as more inconsistent, however, we cannot conclude with certainty that this is explained by what behavior followers expect from their leaders as we did not measure such gender role expectations. Gender role expectations may also differ depending on industry. For example, the positive relationship between leader narcissism and perceived inconsistent leader behavior might be even stronger in more stereotypical female industries (e.g., healthcare) as compared to stereotypical male industries (e.g., finance). We would advise future researchers to look into these underlying mechanisms.

Third, inconsistent behavior should be also investigated over time as a specific display of behavior will be perceived as inconsistent when differing from behavior displayed earlier in time. Therefore, in addition to studying inconsistent behavior cross-sectionally, we would encourage future researchers to look into ways of studying inconsistent leader behaviors longitudinally, for example through experience sampling.

Next, we used a new measure of inconsistent leader behavior, thereby advancing research. However, we encourage researchers to further look into our new scale and further test and extend it. Future research on different dimensions of inconsistent leader behavior could yield compelling insights regarding whether or not some dimensions (e.g., relation-oriented behaviors) send more inconsistent cues than others (e.g., task-oriented behaviors). Furthermore, it would be interesting to take also follower personality into account when looking into the effects of inconsistent leader behavior to see who is better able to deal well with an inconsistent leader and which individuals suffer most.

Further, as we measured performance as a leader rating, it may be possible that this rating is biased by the quality of LMX, where leaders see the followers with whom they have high LMX as performing well. That said, the 0.22 correlation is very similar to the 0.24 overall correlation found in a meta-analysis between LMX and objective measures of performance (see Martin et al., 2016). Also, the rating of follower performance might be biased by leader’s narcissism, potentially in combination with LMX: narcissistic leaders “punish” followers with whom they have low LMX through lower performance evaluations. While we collected multi-source data and did not find a significant correlation between leader narcissism and leader’s rating of follower performance, we did find a significant positive correlation between LMX and follower performance. To avoid this potential bias, future research should collect objective data on follower performance or use performance ratings from different sources (e.g., 360-degree feedback or peer evaluations).

Also, in this study, we focused on the moderating effect of LMX on the relationship between perceived inconsistent leader behavior and follower performance. However, followers perceiving their leader to display inconsistent behavior might in turn like the leader less (i.e., a decrease in the quality of the relationship between leader and follower) and may lower their job performance, which would suggest that LMX mediates the relationship between perceived inconsistent leader behavior and follower performance. Even though we acknowledge that there might be a direct relationship between perceived inconsistent leader behavior and LMX that is most likely negative, we were especially interested in studying the potential buffering effect of LMX. Such focus on the quality of LMX as a moderator is theoretically supported by earlier research showing that LMX influences the link between leader behavior and follower reactions to this behavior (Piccolo and Colquitt, 2006; Michel and Tews, 2016). However, it would still be interesting to study the direct relationship between perceptions of inconsistent leader behavior and LMX. Also, future research could study the potential pathways through which leader narcissism negatively affects LMX (e.g., because narcissistic leaders might generally show less prosocial behavior toward followers).

Finally, it would be of interest to find out whether leader inconsistency can possibly also have positive outcomes. Literature suggests that (narcissistic) leaders strategically act in ways that imply low self-control, because power is associated with the freedom to act according to one’s own volition (Hart et al., 2017). Studies suggest that violating (social) norms indeed fuels perception of power (Van Kleef et al., 2011, 2012). It might be that the agentic traits related to narcissism are perceived as powerful and dominant for male narcissistic leaders, but not or less so for women. This forms an interesting area of research.

## Conclusion

Leader narcissism is evaluated both positively and negatively. Our research provides an explanation for a negative effect of leader narcissism on follower performance by showing that female narcissistic leaders tend to be perceived to show inconsistent behavior, and such behavior relates negatively with performance for followers who have a low-quality relationship with their leader. These results highlight that leaders being perceived as displaying behavioral inconsistency can be a problem, and that gender is an important factor to consider for further studies on this topic.

## Data Availability Statement

The raw data supporting the conclusions of this article will be made available by the authors, without undue reservation.

## Ethics Statement

The studies involving human participants were reviewed and approved by Economics & Business Ethics Committee (EBEC). The patients/participants provided their written informed consent to participate in this study.

## Author Contributions

EG, AH, DH, and FB contributed to conception and design of the study. EG and AH performed the statistical analysis. EG wrote the original draft. All authors contributed to the article and approved the submitted version.

## Conflict of Interest

The authors declare that the research was conducted in the absence of any commercial or financial relationships that could be construed as a potential conflict of interest.

## Publisher’s Note

All claims expressed in this article are solely those of the authors and do not necessarily represent those of their affiliated organizations, or those of the publisher, the editors and the reviewers. Any product that may be evaluated in this article, or claim that may be made by its manufacturer, is not guaranteed or endorsed by the publisher.
